# Supplementary material for: Role for the flagellum attachment zone in Leishmania anterior cell tip morphogenesis
Source: PLoS Pathog. 2020 Oct 22;16(10):e1008494. doi: 10.1371/journal.ppat.1008494 (PMC7608989; doi:10.1371/journal.ppat.1008494)
Supplement: S1 Fig — (A) Confirmation of FAZ2 gene deletion. gDNA from 4 null mutant clones and the parental cells was analysed by PCR. PCR confirmed that FAZ2 ORF was no longer present in the null mutant clones (1–3) and that the resistance markers had integrated correctly in clones (1–3). The neomycin resistance gene had not correctly integrated into clone 4 and this clone was discarded. FAZ2 null mutant clone 1 was used for all subsequent experiments. The lower less distinct band on the gel (*) is likely be non-specific amplification of primer dimers. (B) Western blot confirming expression and expected size (174 kDa) of Ty-mChFP::FAZ2 using the BB2 antibody. The SMP1::eGFP-Ty and BB2 cross reacting band acted as a loading control. (C, D) Measurement of cell body length and width for parental, FAZ2 null mutant and FAZ2 add back cells. These measurements were done independently 3 times on at least 50 1K1N cells. The mean of each replicate is plotted as a circle with the mean and standard deviation of these individual means plotted as black lines. (E) Measurement of flagellum length for parental, FAZ2 null mutant and FAZ2 add back cells. These measurements were done independently 3 times on at least 100 1K1N cells. The mean of each replicate is plotted as a circle with the mean and standard deviation of these individual means plotted as black lines. (PDF) [file ppat.1008494.s001.pdf]

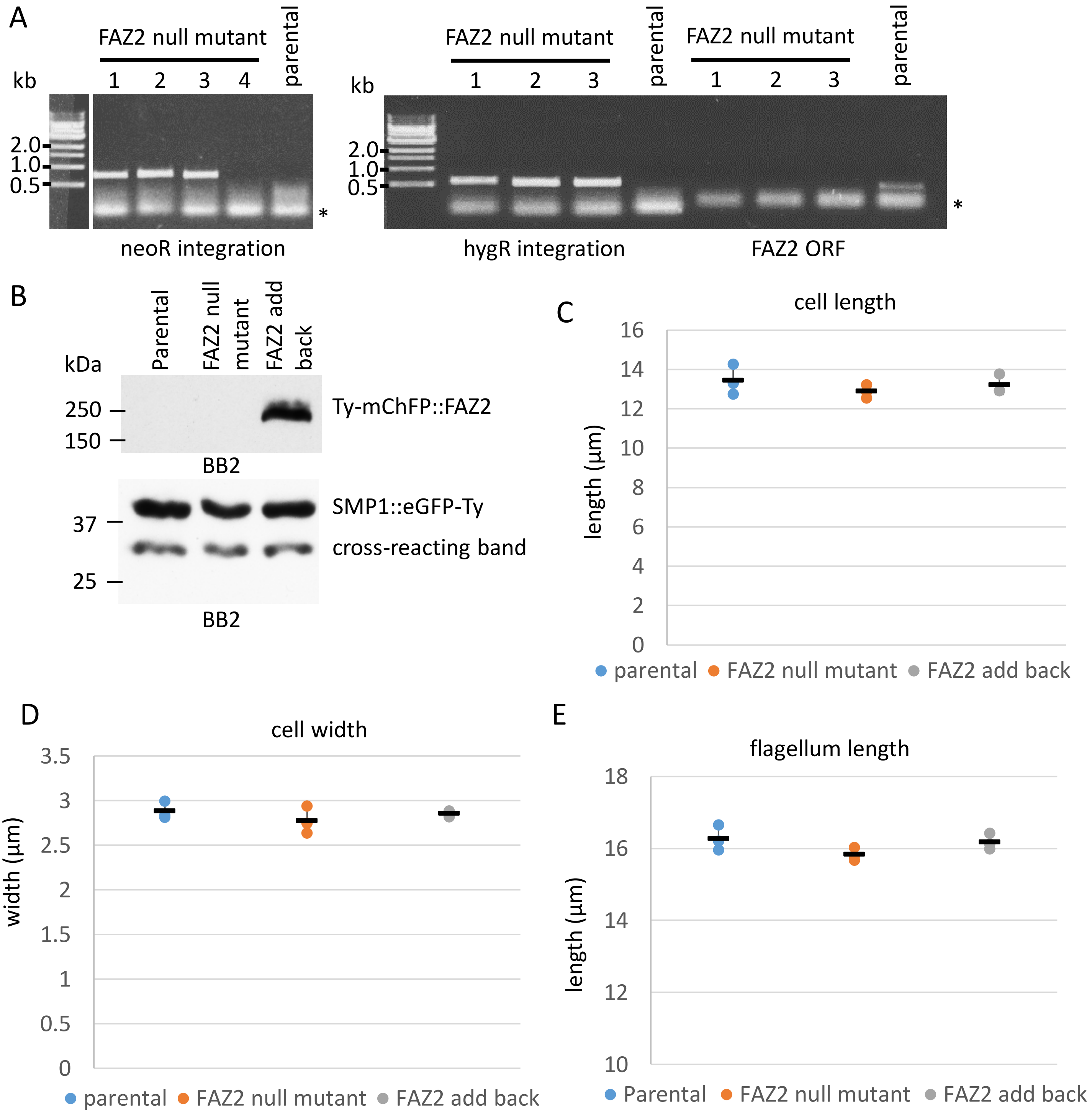

S1 Fig (A) Confirmation of FAZ2 gene deletion. gDNA from 4 null mutant clones and the parental cells was analysed by PCR. PCR confirmed that FAZ2 ORF was no longer present in the null mutant clones (1-3) and that the resistance markers had integrated correctly in clones (1-3). The neomycin resistance gene had not correctly integrated into clone 4 and this clone was discarded. FAZ2 null mutant clone 1 was used for all subsequent experiments. The lower less distinct band on the gel (*) is likely be non-specific amplification of primer dimers. (B) Western blot confirming expression and expected size (174 kDa) of Ty-mChFP::FAZ2 using the BB2 antibody. The SMP1::eGFP-Ty and BB2 cross reacting band acted as a loading control. (C, D) Measurement of cell body length and width for parental, FAZ2 null mutant and FAZ2 add back cells. These measurements were done independently 3 times on at least 50 1K1N cells. The mean of each replicate is plotted as a circle with the mean and standard deviation of these individual means plotted as black lines. (E) Measurement of flagellum length for parental, FAZ2 null mutant and FAZ2 add back cells. These measurements were done independently 3 times on at least 100 1K1N cells. The mean of each replicate is plotted as a circle with the mean and standard deviation of these individual means plotted as black lines.
