# Supplementary material for: Role for the flagellum attachment zone in Leishmania anterior cell tip morphogenesis
Source: PLoS Pathog. 2020 Oct 22;16(10):e1008494. doi: 10.1371/journal.ppat.1008494 (PMC7608989; doi:10.1371/journal.ppat.1008494)
Supplement: S2 Fig — (A) Confirmation of FAZ2 gene deletion. gDNA from 4 null mutant clones and the parental cells was analysed by PCR. (B) Quantitation of cell types seen in culture for C9/T7 and FAZ2 null mutant clones. This experiment was performed once and for each cell line ≥84 cells were counted. (C) Cell cycle category counts for C9/T7 and FAZ2 null mutant clones. F–flagellum, K–kinetoplast, N–nucleus, F to F–two cells connected via their flagella. This experiment was performed once and for each cell line ≥110 cells were counted. (D) Measurement of the distance between the kinetoplast and the anterior end of the cell body for C9/T7 and FAZ2 null mutant clones. This experiment was performed once, each measurement is a coloured circle with the mean and s.d. plotted as black lines. For each cell line ≥62 cells were measured. (PDF) [file ppat.1008494.s002.pdf]

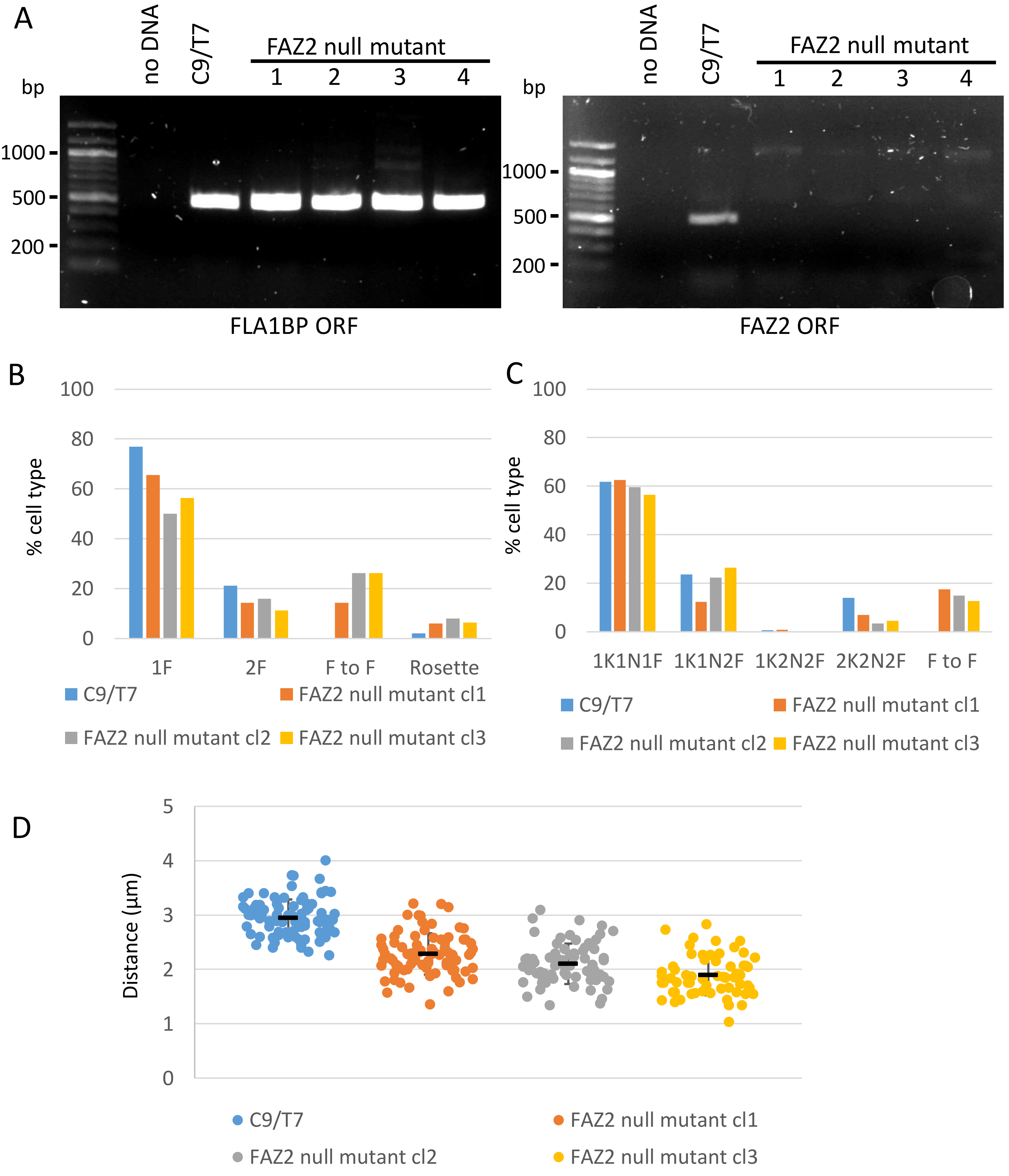

S2 Fig (A) Confirmation of FAZ2 gene deletion. gDNA from 4 null mutant clones and the parental cells was analysed by PCR. (B) Quantitation of cell types seen in culture for C9/T7 and FAZ2 null mutant clones. This experiment was performed once and for each cell line ≥84 cells were counted. (C) Cell cycle category counts for C9/T7 and FAZ2 null mutant clones. F – flagellum, K – kinetoplast, N – nucleus, F to F – two cells connected via their flagella. This experiment was performed once and for each cell line ≥110 cells were counted. (D) Measurement of the distance between the kinetoplast and the anterior end of the cell body for C9/T7 and FAZ2 null mutant clones. This experiment was performed once, each measurement is a coloured circle with the mean and s.d. plotted as black lines. For each cell line ≥62 cells were measured.
