# Supplementary material for: Role for the flagellum attachment zone in Leishmania anterior cell tip morphogenesis
Source: PLoS Pathog. 2020 Oct 22;16(10):e1008494. doi: 10.1371/journal.ppat.1008494 (PMC7608989; doi:10.1371/journal.ppat.1008494)
Supplement: S3 Fig — Location of Leishmania parasites within infected sand flies at 1–2 and 6–8 days post blood meal. Stacked columns indicate the percentage of infected sand flies with parasites in various locations within the sand fly. FAZ2 null mutant was unable to migrate to the stomodeal valve. Percentage of infected flies for each cell line is indicated above each column. This is the combined data from two independent sand fly infection experiments. (PDF) [file ppat.1008494.s003.pdf]

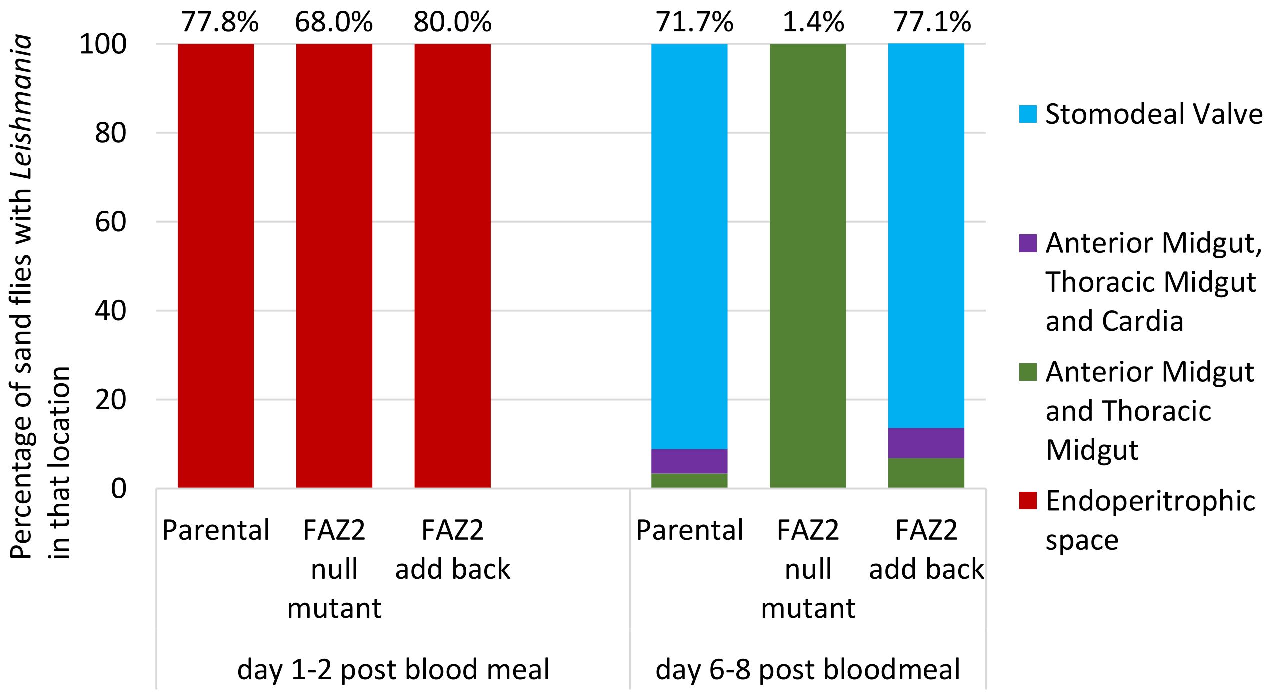

S3 Fig Migration of *Leishmania* in sand fly gut. Location of *Leishmania* parasites within infected sand flies at 1-2 and 6-8 days post blood meal. Stacked columns indicate the percentage of infected sand flies with parasites in various locations within the sand fly. FAZ2 null mutant was unable to migrate to the stomodeal valve. Percentage of infected flies for each cell line is indicated above each column. This is the combined data from two independent sand fly infection experiments.
