# Supplementary material for: Role for the flagellum attachment zone in Leishmania anterior cell tip morphogenesis
Source: PLoS Pathog. 2020 Oct 22;16(10):e1008494. doi: 10.1371/journal.ppat.1008494 (PMC7608989; doi:10.1371/journal.ppat.1008494)
Supplement: S4 Fig — (A) Swimming tracks from videomicroscopy of parental, FAZ2 null mutant and FAZ2 add back cells. Cells were imaged for 61 seconds with 512 images taken. Scale bar is 50 μm. (B) Histograms of the mean speed for parental, FAZ2 null mutant and FAZ2 add back cells for all tracks imaged and for 50 1F cells and 50 F to F cells. (C) Histograms of the directional persistence for parental, FAZ2 null mutant and FAZ2 add back cells for all tracks imaged and for 50 1F cells and 50 F to F cells. The histograms and tracks are representative of two independent replicates. (PDF) [file ppat.1008494.s004.pdf]

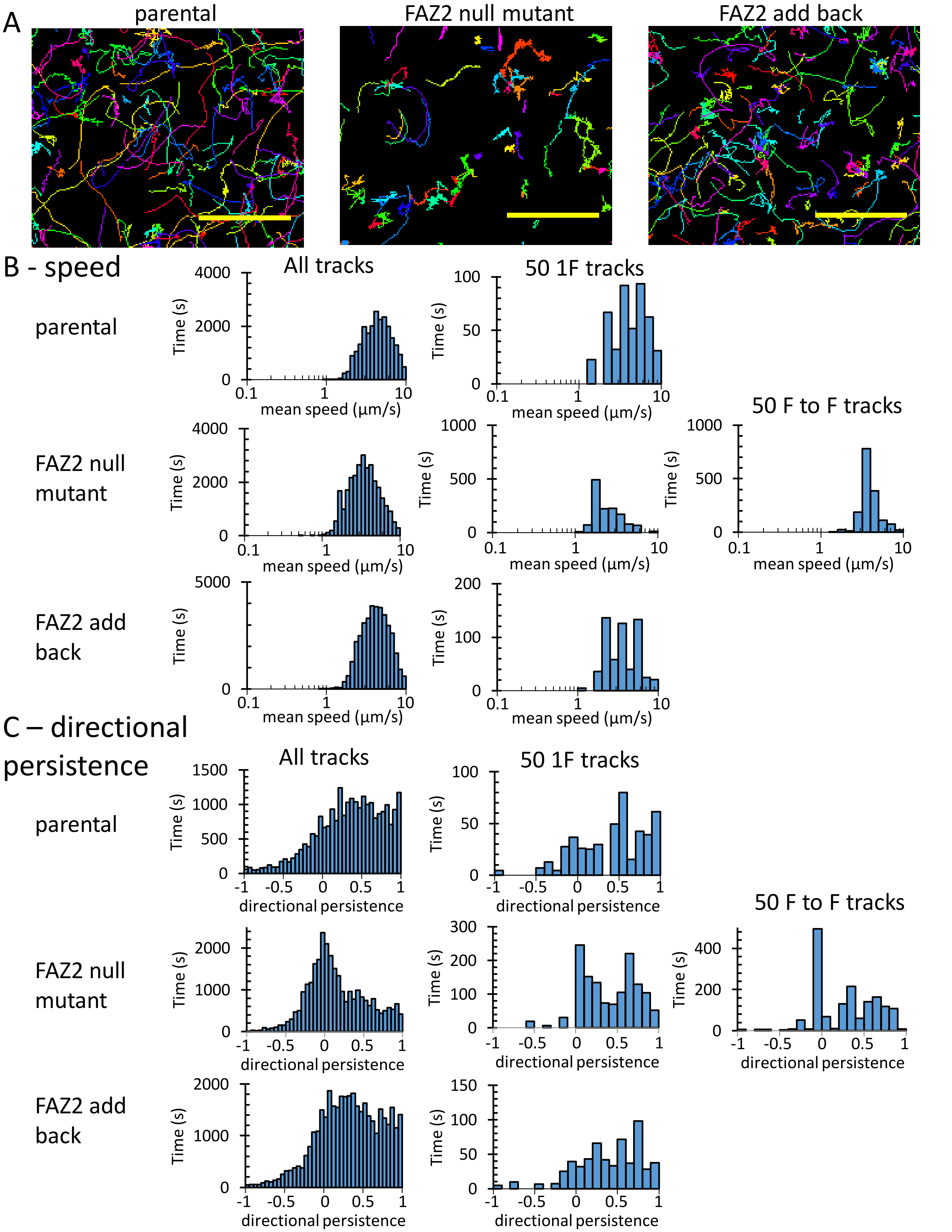

S4 Fig (A) Swimming tracks from videomicroscopy of parental, FAZ2 null mutant and FAZ2 add back cells. Cells were imaged for 61 seconds with 512 images taken. Scale bar is 50 µm. (B) Histograms of the mean speed for parental, FAZ2 null mutant and FAZ2 add back cells for all tracks imaged and for 50 1F cells and 50 F to F cells. (C) Histograms of the directional persistence for parental, FAZ2 null mutant and FAZ2 add back cells for all tracks imaged and for 50 1F cells and 50 F to F cells. The histograms and tracks are representative of two independent replicates.
