# Supplementary material for: Role for the flagellum attachment zone in Leishmania anterior cell tip morphogenesis
Source: PLoS Pathog. 2020 Oct 22;16(10):e1008494. doi: 10.1371/journal.ppat.1008494 (PMC7608989; doi:10.1371/journal.ppat.1008494)
Supplement: S5 Fig — (A) Images of axenic amastigotes of parental, FAZ2 null mutant and FAZ2 add back cells expressing SMP1::eGFP-Ty. Scale bar is 5 μm. (B) Leishmania macrophage infections. Growth curve of parental, FAZ2 null mutant and FAZ2 add back cells to stationary phase—average of 3 replicates, mean ± s.d is plotted. (C, D) Proportion of infected macrophages and the number of Leishmania per infected macrophage at 0, 24, 48, 72 hours post infection—0 h time point is after 2 hours of infection and removal of cells not taken up. For each time point between 487–1074 macrophages were analysed. Mean ± s.d. for 3 replicates is shown. (PDF) [file ppat.1008494.s005.pdf]

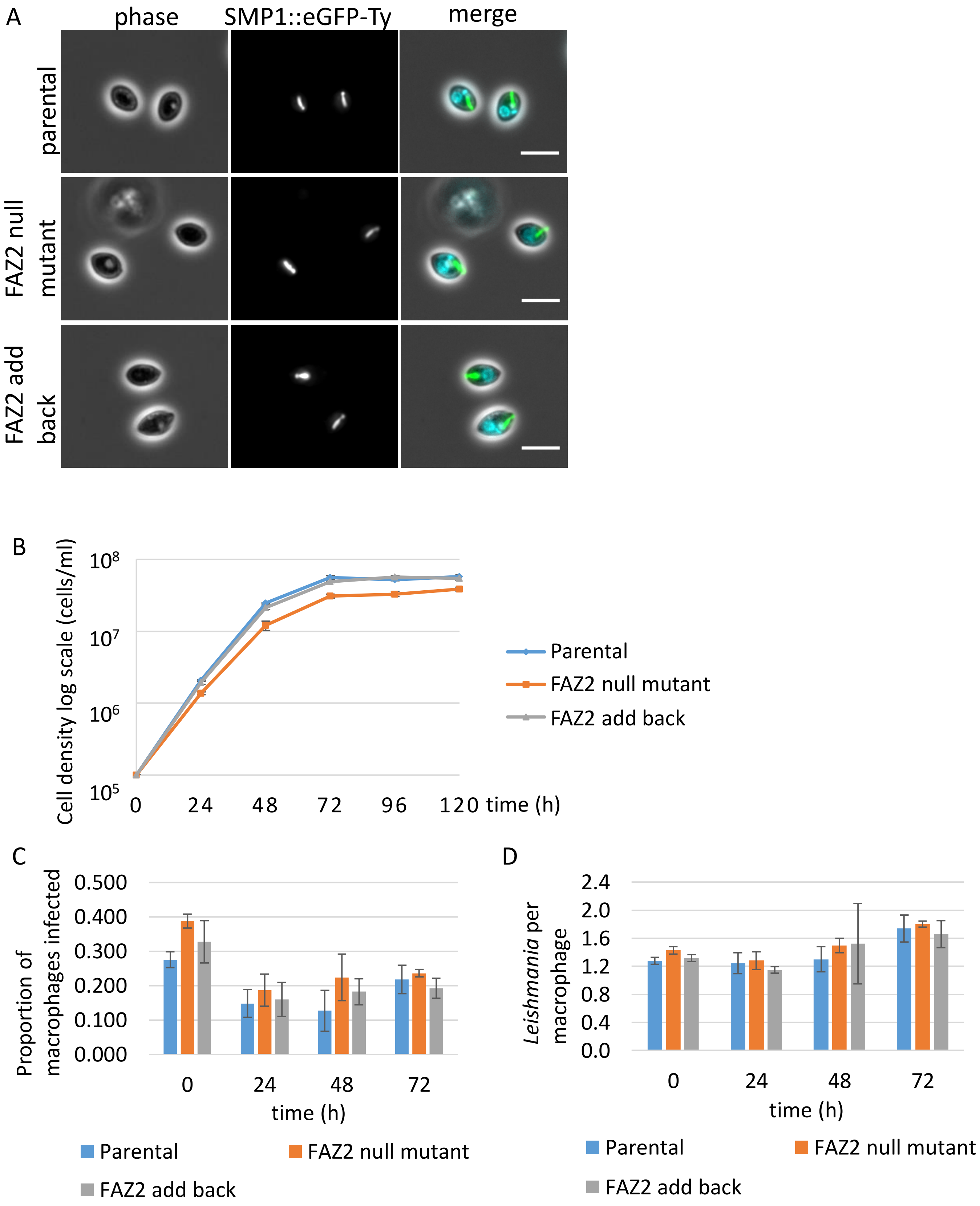

S5 Fig (A) Images of axenic amastigotes of parental, FAZ2 null mutant and FAZ2 add back cells expressing SMP1::eGFP-Ty. Scale bar is 5 μm. (B) *Leishmania* macrophage infections. Growth curve of parental, FAZ2 null mutant and FAZ2 add back cells to stationary phase - average of 3 replicates, mean ± s.d is plotted. (C, D) Proportion of infected macrophages and the number of *Leishmania* per infected macrophage at 0, 24, 48, 72 hours post infection - 0 h time point is after 2 hours of infection and removal of cells not taken up. For each time point between 487-1074 macrophages were analysed. Mean ± s.d. for 3 replicates is shown.
